# Impact of the 2022 pulmonary hypertension definition on haemodynamic classification and mortality in patients with aortic stenosis undergoing valve replacement

**DOI:** 10.1093/ehjopen/oeae037

**Published:** 2024-05-29

**Authors:** Micha T Maeder, Lukas Weber, Susanne Pohle, Joannis Chronis, Florent Baty, Johannes Rigger, Martin Brutsche, Philipp Haager, Hans Rickli, Roman Brenner

**Affiliations:** Department of Cardiology, Kantonsspital St. Gallen, Rorschacherstrasse 95, CH-9007 St. Gallen, Switzerland; Departement of Medicine, University of Basel, Klingelbergstrasse 61, CH-4056 Basel, Switzerland; Department of Cardiology, Kantonsspital St. Gallen, Rorschacherstrasse 95, CH-9007 St. Gallen, Switzerland; Lung Center, Kantonsspital St. Gallen, Rorschacherstrasse 95, CH-9007 St. Gallen, Switzerland; Department of Cardiology, Kantonsspital St. Gallen, Rorschacherstrasse 95, CH-9007 St. Gallen, Switzerland; Lung Center, Kantonsspital St. Gallen, Rorschacherstrasse 95, CH-9007 St. Gallen, Switzerland; Department of Cardiology, Kantonsspital St. Gallen, Rorschacherstrasse 95, CH-9007 St. Gallen, Switzerland; Departement of Medicine, University of Basel, Klingelbergstrasse 61, CH-4056 Basel, Switzerland; Lung Center, Kantonsspital St. Gallen, Rorschacherstrasse 95, CH-9007 St. Gallen, Switzerland; Department of Cardiology, Kantonsspital St. Gallen, Rorschacherstrasse 95, CH-9007 St. Gallen, Switzerland; Department of Cardiology, Kantonsspital St. Gallen, Rorschacherstrasse 95, CH-9007 St. Gallen, Switzerland; Department of Cardiology, Kantonsspital St. Gallen, Rorschacherstrasse 95, CH-9007 St. Gallen, Switzerland

**Keywords:** Pulmonary hypertension, Aortic stenosis, Definition, Pulmonary artery pressure, Pulmonary vascular resistance, Pulmonary artery compliance

## Abstract

**Aims:**

With the 2022 pulmonary hypertension (PH) definition, the mean pulmonary artery pressure (mPAP) threshold for any PH was lowered from ≥25 to >20 mmHg, and the pulmonary vascular resistance (PVR) value to differentiate between isolated post-capillary PH (IpcPH) and combined pre- and post-capillary PH (CpcPH) was reduced from >3 Wood units (WU) to >2 WU. We assessed the impact of this change in the PH definition in aortic stenosis (AS) patients undergoing aortic valve replacement (AVR).

**Methods and results:**

Severe AS patients (*n* = 503) undergoing pre-AVR cardiac heart catheterization were classified according to both the 2015 and 2022 definitions. The post-AVR mortality [median follow-up 1348 (interquartile range 948–1885) days] was assessed. According to the 2015 definition, 219 (44% of the entire population) patients had PH: 63 (29%) CpcPH, 125 (57%) IpcPH, and 31 (14%) pre-capillary PH. According to the 2022 definition, 321 (+47%) patients were diagnosed with PH, and 156 patients (31%) were re-classified: 26 patients from no PH to IpcPH, 38 from no PH to pre-capillary PH, 38 from no PH to unclassified PH, 4 from pre-capillary PH to unclassified PH, and 50 from IpcPH to CpcPH (CpcPH: +79%). With both definitions, only the CpcPH patients displayed increased mortality (hazard ratios ≈ 4). Among the PH-defining haemodynamic components, PVR was the strongest predictor of death.

**Conclusion:**

In severe AS, the application of the 2022 PH definition results in a substantially higher number of patients with any PH as well as CpcPH. With either definition, CpcPH patients have a significantly increased post-AVR mortality.

## Graphical Abstract

**Graphical abstract:**
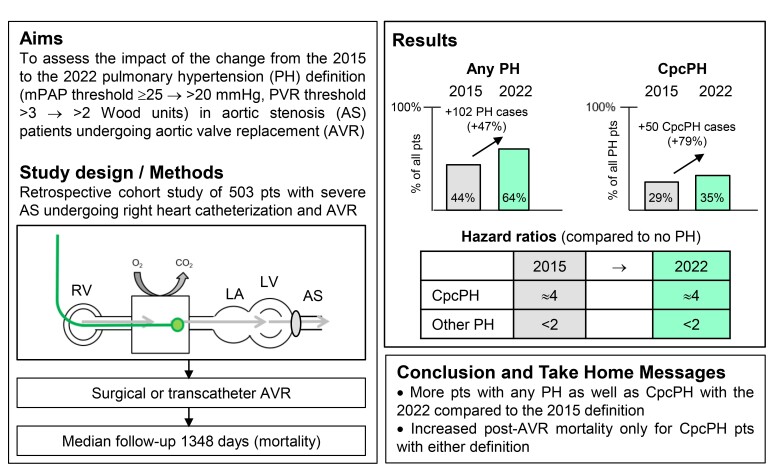
AS, aortic stenosis; CO_2_, carbon dioxide; CpcPH, combined pre- and post-capillary pulmonary hypertension; IpcPH, isolated post-capillary pulmonary hypertension; LA, left atrium; LV, left ventricle; mPAP, mean pulmonary artery pressure; mPAWP, mean pulmonary artery wedge pressure; O_2_, oxygen; PVR, pulmonary vascular resistance; PH, pulmonary hypertension.

## Introduction

The 2022 guidelines of the European Society of Cardiology (ESC)/European Respiratory Society (ERS) on pulmonary hypertension (PH) include a modified PH definition,^1^ which substantially differs from the previous 2015 ESC/ERS definition.^2^ First, the threshold for the mean pulmonary artery pressure (mPAP) for the definition of any PH has been lowered from ≥25 to >20 mmHg (i.e. a reduction by 4 mmHg). Second, the threshold for the pulmonary vascular resistance (PVR) to differentiate between combined pre- and post-capillary PH (CpcPH) and isolated post-capillary PH (IpcPH) has been reduced from >3 to >2 Wood units (WU), while the threshold for the mean pulmonary artery wedge pressure (mPAWP) to differentiate between pre-capillary and post-capillary PH has been left unchanged (≤15 vs. >15 mmHg). Third, the definition of pre-capillary PH is not only based on an mPAWP ≤ 15 mmHg anymore, but also requires an elevated PVR, i.e. >2 WU. Fourth, there is an explicitly defined new haemodynamic category of patients with ‘unclassified PH’, i.e. those with mPAP > 20 mmHg and the combination of an mPAWP ≤ 15 mmHg and a PVR ≤ 2 WU.^1^ We had previously reported on the application of the 2018 PH World Symposium proposal compared with the 2015 ESC/ERS definition, where we found a 2% increase in the number of patients with any PH and a re-classification rate of 11%.^3^ However, the 2022 PH definition also substantially differs from the 2018 PH World Symposium proposal,^4^ the key differences being the new PVR criterion to define pre-capillary PH and CpcPH respectively (>2 WU vs. >3 WU) and the explicit definition of ‘unclassified PH’.

There are still little data on the impact of these substantial changes in the PH definition in patient cohorts seen in clinical practice. A recent UK national study in a population with predominantly pre-capillary PH has confirmed the prognostic importance of mildly elevated mPAP (20–24 mmHg) and PVR (2–3 WU) values.^5^ However, overall post-capillary PH is by far the most common form of PH,^1^ and the role of the current PH definition in this setting also needs a detailed description. The few available data indicate that more patients are diagnosed with CpcPH because the diagnostic mPAP and PVR thresholds are lower in the 2022 than in the 2015 definition.^6–9^ However, studies are controversial regarding the question whether CpcPH according to the newer, less restrictive definition still identifies a group with particular poor prognosis in populations with predominant group 2 PH.^6,9,10^

The aim of the present study was to describe the impact of the 2022 ESC/ERS definition on the PH classification and its prognostic implications in patients with aortic stenosis (AS) undergoing valve replacement.

## Methods

### Study population

This is a retrospective analysis of prospectively and systematically collected cardiac catheterization data in 503 consecutive patients with severe AS undergoing cardiac catheterization prior to AVR in a single centre between January 2011 and January 2016.^11^ At our institution, all patients with severe AS undergo right heart catheterization and coronary angiography on a routine basis prior to AVR. Accordingly, this is an unselected all-comers cohort of AS patients evaluated prior to AVR. Complete data on mPAP, mPAWP, and PVR were available for all patients so that classification could be performed according to both the 2015^1^ and 2022 guidelines.^2^ The study was approved by the Ethics Committee of the Canton of St. Gallen (project number 2016-02113, date of approval: 3 January 2017). Owing to its retrospective design, a waiver of consent was granted for this study.

### Cardiac catheterization

Procedures were generally (>95%) performed in the morning in the fasting state and after withholding loop diuretics and renin–angiotensin system inhibitors. Patients underwent coronary angiography using five or six French catheters via the femoral or radial artery and right heart catheterization using six French Swan Ganz catheters via femoral or brachial access. The midthoracic level was used as zero reference point. Right atrial pressure, right ventricular pressure, pulmonary artery pressure, and pulmonary artery wedge pressure were measured. The wedge position was confirmed by fluoroscopy and waveform analysis. Measurements were obtained at end-expiration, the mPAWP was calculated over the entire cardiac cycle, and v-waves were included to determine mPAWP. This practice leads to higher values compared with the measurement of the end-diastolic pulmonary artery wedge pressure. However, for the estimation of the impact of the left heart contribution to pulmonary pressures and the calculation of PVR, the mPAWP is preferred.^12^ In patients with atrial fibrillation, at least five cardiac cycles were used to assess pulmonary artery pressure and pulmonary artery wedge pressure. Cardiac output was assessed by the indirect Fick method based on blood gases, with blood samples taken in duplicate via arterial access and the pulmonary artery. The transpulmonary gradient was calculated as the difference between mPAP and mPAWP and PVR as a transpulmonary gradient divided by cardiac output. Pulmonary artery compliance (PAC) was calculated as the stroke volume divided by the difference of the systolic and diastolic pulmonary artery pressures. If the aortic valve was crossed, the left ventricular end-diastolic pressure (LVEDP) was recorded. All pressure readings were double-checked by the operator using a manual review of the pressure tracings before recording them into the report.

### Haemodynamic definitions

Patients were classified according to both the 2015^2^ and 2022 ESC/ERS guidelines.^1^ For the 2015 definition, we did not use the diastolic pressure gradient criterion because this leads to unclassifiable patients,^13^ diastolic pressure gradient values are often negative, and data on the prognostic impact of the diastolic pressure gradient are conflicting.^14^ Thus, according to the 2015 ESC/ERS guidelines,^2^ any PH was defined as mPAP ≥ 25 mmHg; IpcPH was defined as mPAP ≥ 25 mmHg, mPAWP > 15 mmHg, and PVR ≤ 3 WU; CpcPH was defined as mPAP ≥ 25 mmHg, mPAWP > 15 mmHg, and PVR > 3 WU; and pre-capillary PH was defined as mPAP ≥ 25 mmHg and mPAWP ≤ 15 mmHg (i.e. no PVR criterion). According to the 2022 guidelines,^1^ any PH was defined as mPAP > 20 mmHg; IpcPH was defined as mPAP > 20 mmHg, mPAWP >15 mmHg, and PVR ≤2 WU; CpcPH was defined as mPAP > 20 mmHg, mPAWP > 15 mmHg, and PVR > 2 WU; and pre-capillary PH was defined as mPAP > 20 mmHg, mPAWP ≤ 15 mmHg, and PVR > 2 WU. Patients with mPAP > 20 mmHg, mPAWP ≤ 15 mmHg, and PVR ≤ 2 WU were allocated to the new category of ‘unclassified PH’.^1^

### Echocardiography

In all patients, an echocardiogram was carried out prior to performing cardiac catheterization, and it was used as a basis for the referral. Echocardiograms were performed by experienced cardiologists in line with contemporary guidelines, but without adhering to a specified protocol. The data were retrospectively retrieved from patients’ medical reports.

### Follow-up

All patients underwent surgical (SAVR; 71%) or transcatheter (TAVR; 29%) AVR following a median interval of 21 days (12–35) post-catheterization. Information on the long-term follow-up was obtained from patients, general practitioners, and hospital or practice cardiologists. The endpoint was all-cause mortality.

### Statistical analysis

Categorical data were presented as numbers and percentages and continuous data as mean ± standard deviation or median (interquartile range), as appropriate. Patients from different haemodynamic PH categories and those without PH according to the 2015 and 2022 definitions were compared using χ^2^ tests, analysis of variance, or Kruskal–Wallis tests, as appropriate. Patients who were re-classified by the 2022 definition compared with the 2015 definition and those who were not were compared using χ^2^ tests, unpaired *t*-tests, or Mann–Whitney *U*-tests. Survival of patients from different haemodynamic categories was investigated using Kaplan–Meier estimates and compared using log-rank tests. Cox proportional hazards regression was applied to describe the association between variables of interest and mortality. A *P* value < 0.05 was considered statistically significant. Analyses were performed using SPSS Statistical Package Version 20.0 (SPSS Inc., Chicago, IL, USA).

## Results

### Study population

The mean age of the 503 patients was 74 ± 10 years, and 290 (58%) were male. The mean indexed aortic valve area was 0.42 ± 0.12 cm^2^/m^2^, and the mean left ventricular ejection fraction was 57 ± 12%. The mean mPAP, mPAWP, and PVR were 25 ± 10 mmHg, 16 ± 8 mmHg, and 2.1 ± 1.5 WU, respectively.

### Classification according to the 2015 definition

**Figure 1:**
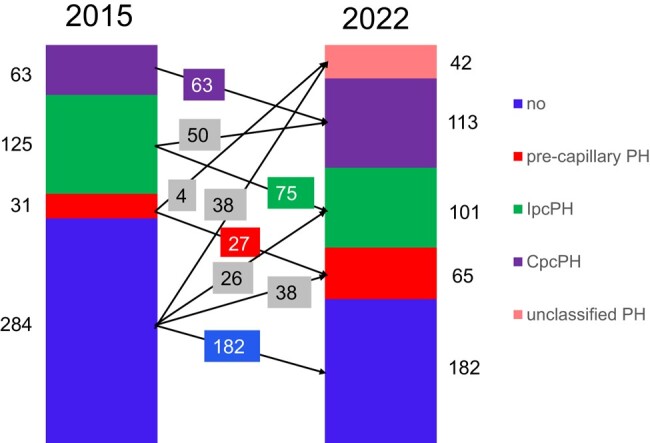
Bar graph showing the proportion of patients (*n* = 503) with combined pre- and post-capillary pulmonary hypertension (CpcPH), isolated post-capillary PH (IpcPH), pre-capillary PH, unclassified PH, and without PH according to the 2015 and 2022 definitions and the re-classification steps.

According to the 2015 ESC/ERS guidelines, there were 219 (44% of the entire population) patients with any PH, 63 (29% of patients with PH) of whom had CpcPH, 125 (57% of patients with PH) had IpcPH, and 31 (14% of patients with PH) had pre-capillary PH, whereas 284 (56% of the entire population) patients had no PH (*Figure 1*). Clinical characteristics, echocardiographic findings, and invasive haemodynamics of patients with CpcPH, IpcPH, pre-capillary PH, and those without PH according to the 2015 definition are shown in Supplementary material online, *Tables S1* and *S2*.

### Classification according to the 2022 definition

According to the 2022 definition, 321 [64% of the entire population; + 102 (+47% compared with the number of PH patients according to the 2015 definition)] patients had any PH: 113 (35% of patients with PH; +50) had CpcPH, 101 (31% of patients with PH; −24) had IpcPH, 65 (20% of patients with PH; +34) had pre-capillary PH, and 42 (13% of patients with PH; +42; new category) had unclassified PH, whereas 182 (55% of the entire population; −102) patients had no PH (*Figure 1*). Clinical characteristics, echocardiographic findings, and invasive haemodynamics of patients with CpcPH, IpcPH, pre-capillary PH, unclassified PH, and those without PH according to the 2022 definition are shown in *Tables 1* and *2*. There were several significant differences between the haemodynamic groups. In particular, patients with CpcPH had the smallest indexed aortic valve area and the lowest tricuspid annular plane systolic excursion.

**Table 1:** Clinical characteristics of the haemodynamic groups according to the 2022 definition

	CpcPH (*n* = 113)	IpcPH (*n* = 101)	Pre-capillary PH (*n* = 65)	Unclassified PH (*n* = 42)	No PH (*n* = 182)	*P* value
Age (years)	77 ± 9	74 ± 10	77 ± 7	76 ± 8	71 ± 11	<0.001
Sex (male)	54 (48%)	68 (67%)	34 (52%)	30 (71%)	104 (57%)	0.01
Body mass index (kg/m^2^)	28.1 ± 5.5	29.0 ± 5.5	27.7 ± 5.5	28.5 ± 4.2	27.0 ± 4.5	0.03
eGFR (mL/min/1.73 m^2^)	60 ± 21	66 ± 18	65 ± 18	62 ± 18	71 ± 16	<0.001
Haemoglobin (g/L)	130 ± 19	137 ± 15	133 ± 22	139 ± 15	138 ± 16	0.001
Albumin (g/L)	37 ± 5	38 ± 3	38 ± 4	38 ± 3	39 ± 6	0.002
Sodium (mmol/L)	137 ± 4	138 ± 3	138 ± 3	139 ± 3	137 ± 3	0.14
Potassium (mmol/L)	4.0 ± 0.5	4.0 ± 0.5	4.0 ± 0.4	4.0 ± 0.5	4.0 ± 0.5	0.64
Diabetes	25 (22%)	30 (30%)	16 (25%)	7 (17%)	27 (15%)	0.04
Insulin-dependent	8 (7%)	6 (6%)	4 (6%)	1 (2%)	3 (2%)	0.15
Stroke	8 (7%)	7 (7%)	5 (8%)	1 (2%)	9 (5%)	0.72
Chronic obstructive pulmonary disease	15 (13%)	8 (8%)	17 (26%)	4 (10%)	15 (8%)	0.002
GOLD stage 1	4	1	0	0	2	
GOLD stage 2	7	6	10	2	10	
GOLD stage 3	3	1	7	2	3	
GOLD stage unknown	1	0	0	0	0	
CPAP therapy	0	1 (1%)	0	0	1 (<1%)	0.75
Previous PCI	14 (12%)	8 (8%)	7 (11%)	5 (12%)	12 (7%)	0.46
Previous CABG	8 (7%)	9 (9%)	4 (6%)	2 (5%)	4 (2%)	0.14
FEV1 (% predicted)	79 ± 18	86 ± 19	76 ± 23	89 ± 21	94 ± 18	<0.001
**Heart rhythm** ^a^						<0.001
Sinus rhythm	83 (74%)	85 (84%)	60 (92%)	39 (93%)	169 (93%)	
Atrial fibrillation	25 (22%)	13 (13%)	4 (6%)	2 (5%)	6 (3%)	
Pacemaker	5 (4%)	3 (3%)	1 (2%)	1 (2%)	7 (4%)	
Heart rate (b.p.m.)	74 ± 15	71 ± 13	70 ± 14	68 ± 10	66 ± 11	<0.001
**Medication**						
Oral anticoagulation	41 (36%)	17 (17%)	7 (11%)	7 (17%)	22 (12%)	<0.001
Aspirin	59 (52%)	65 (64%)	42 (65%)	30 (71%)	112 (62%)	0.17
Loop diuretics	88 (78%)	60 (59%)	31 (48%)	15 (36%)	55 (30%)	<0.001
Beta-blocker	60 (53%)	53 (52%)	36 (55%)	19 (45%)	70 (38%)	0.04
ACEI/ARB	58 (51%)	62 (61%)	42 (65%)	21 (50%)	94 (52%)	0.20
Digoxin	18 (16%)	6 (6%)	3 (5%)	1 (2%)	4 (2%)	<0.001
Spironolactone	13 (12%)	4 (4%)	2 (3%)	0	6 (3%)	0.07
B-type natriuretic peptide (ng/L)	625 (285–1200)	310(180–530)	161 (76–359)	146 (68–281)	91 (51–209)	<0.001
**Symptoms**						
Dyspnoea NYHA class						<0.001
I	10 (9%)	17 (17%)	13 (20%)	4 (10%)	57 (32%)	
II	45 (40%)	48 (47%)	29 (45%)	29 (69%)	95 (52%)	
III	46 (41%)	32 (32%)	21 (32%)	8 (19%)	26 (14%)	
IV	12 (10%)	4 (4%)	2 (3%)	1 (2%)	4 (2%)	
STS score	4.4 ± 3.1	2.9 ± 2.3	3.1 ± 2.4	2.4 ± 1.5	2.1 ± 1.4	<0.001
**Mode of AVR**						<0.001
Surgical AVR	63 (56%)	66 (65%)	41 (63%)	31 (74%)	160 (88%)	
Transcatheter AVR	50 (44%)	35 (35%)	24 (37%)	11 (26%)	22 (12%)	

Data are given as numbers and percentages, mean ± standard deviation, or median (interquartile range).

ACEI/ARB, angiotensin-converting enzyme inhibitor/angiotensin receptor blocker; AVR, aortic valve replacement; CPAP, continuous positive airway pressure; eGFR, estimated glomerular filtration rate; FEV1, forced expiratory volume within the first second (percent predicted); GOLD, global initiative for chronic obstructive lung disease; NYHA, New York Heart Association; STS, Society of Thoracic Surgeons.

^a^Rhythm at the time of cardiac catheterization.

**Table 2:** Data from echocardiography and cardiac catheterization of haemodynamic groups according to the 2022 classification

	CpcPH (*n* = 113)	IpcPH (*n* = 101)	Pre-capillary PH (*n* = 65)	Unclassified PH (*n* = 42)	No PH (*n* = 182)	*P* value
**Echocardiography**						
Left ventricular end-diastolic diameter (mm)	47 ± 9	47 ± 7	44 ± 7	48 ± 8	45 ± 8	0.01
Indexed left ventricular end-diastolic diameter (mm/m^2^)	26 ± 5	25 ± 3	25 ± 4	24 ± 4	24 ± 4	0.12
Septal wall thickness (mm)	14 ± 3	13 ± 3	13 ± 3	12 ± 3	12 ± 3	0.01
Posterior wall thickness (mm)	11 ± 3	11 ± 3	11 ± 2	12 ± 3	11 ± 2	0.21
Left ventricular mass index (g/m^2^)	126 ± 37	112 ± 31	104 ± 31	110 ± 38	103 ± 36	<0.001
Left ventricular end-diastolic volume index (mL/m^2^)	48 ± 20	46 ± 16	43 ± 16	41 ± 12	43 ± 16	0.16
Left ventricular ejection fraction (%)	52 ± 14	55 ± 13	59 ± 10	61 ± 7	61 ± 10	<0.001
E/e′	22 ± 11	17 ± 8	18 ± 9	16 ± 8	13 ± 5	<0.001
Left atrial diameter (mm)	44 ± 7	43 ± 6	40 ± 7	40 ± 6	38 ± 7	<0.001
Indexed left atrial diameter (mm/m^2^)	24 ± 4	22 ± 3	22 ± 4	20 ± 3	20 ± 4	<0.001
Left atrial area (cm^2^)	27 ± 8	24 ± 6	23 ± 7	22 ± 6	21 ± 6	<0.001
Indexed left atrial area (cm^2^/m^2^)	15 ± 4	13 ± 3	12 ± 4	11 ± 3	11 ± 3	<0.001
Left atrial volume index (mL/m^2^)	52 ± 21	45 ± 14	43 ± 18	36 ± 13	37 ± 12	<0.001
Indexed RV basal diameter (mm/m^2^)	18 ± 4	16 ± 3	17 ± 4	15 ± 4	15 ± 3	<0.001
Indexed right atrial area (cm^2^/m^2^)	10 ± 3	8 ± 3	9 ± 3	8 ± 4	8 ± 2	<0.001
Right atrial volume index (mL/m^2^)	30 ± 16	23 ± 12	24 ± 11	20 ± 7	21 ± 10	<0.001
TAPSE (mm)	19 ± 5	21 ± 5	23 ± 5	22 ± 4	22 ± 5	0.001
Estimated sPAP (mmHg)	48 ± 14	39 ± 10	41 ± 15	36 ± 9	33 ± 8	<0.001
Mean aortic valve gradient (mmHg)	47 ± 19	48 ± 18	48 ± 16	45 ± 18	47 ± 16	0.85
Aortic valve area (cm^2^)	0.72 ± 0.22	0.77 ± 0.21	0.81 ± 0.21	0.85 ± 0.25	0.83 ± 0.25	0.002
Indexed aortic valve area (cm^2^/m^2^)	0.39 ± 0.12	0.40 ± 0.10	0.44 ± 0.12	0.44 ± 0.13	0.45 ± 0.13	<0.001
**Mitral regurgitation**						<0.001
No	24 (21%)	34 (34%)	36 (55%)	23 (55%)	121 (66%)	
Mild	63 (56%)	59 (58%)	22 (34%)	17 (41%)	54 (30%)	
Moderate	22 (19%)	6 (6%)	5 (8%)	1 (2%)	6 (3%)	
Severe	4 (4%)	2 (2%)	2 (3%)	1 (2%)	1 (1%)	
**Coronary artery disease**						0.28
No coronary artery disease	52 (46%)	55 (54%)	31 (48%)	20 (48%)	106 (58%)	
One-vessel disease	16 (14%)	18 (18%)	15 (23%)	7 (17%)	31 (17%)	
Two-vessel disease	22 (20%)	12 (12%)	8 (12%)	4 (9%)	24 (13%)	
Three-vessel disease	23 (20%)	16 (16%)	11 (17%)	11 (26%)	21 (12%)	
**Invasive haemodynamics**						
Mean right atrial pressure (mmHg)	10 ± 4	8 ± 3	6 ± 3	7 ± 2	5 ± 3	<0.001
Right ventricular end-diastolic pressure (mmHg)	12 ± 4	10 ± 3	8 ± 3	8 ± 2	6 ± 3	<0.001
sPAP (mmHg)	59 ± 14	42 ± 8	39 ± 11	36 ± 10	28 ± 5	<0.001
dPAP (mmHg)	24 ± 7	18 ± 5	15 ± 4	13 ± 2	9 ± 3	<0.001
mPAP (mmHg)	39 ± 9	28 ± 5	25 ± 4	22 ± 1	16 ± 3	<0.001
mPAWP (mmHg)	24 ± 6	22 ± 5	12 ± 2	14 ± 1	9 ± 3	<0.001
Transpulmonary gradient (mmHg)	14 ± 5	6 ± 2	13 ± 6	9 ± 2	7 ± 3	<0.001
Pulmonary vascular resistance (Wood units)	3.3 (2.5–4.1)	1.3 (0.9–1.7)	2.6 (2.2–3.1)	1.7 (1.6–1.8)	1.4 (1.0–1.8)	
Pulmonary artery compliance (mL/mmHg)	1.8 ± 0.8	3.2 ± 1.3	3.1 ± 1.1	3.7 ± 1.0	4.4 ± 2.1	<0.001
Left ventricular end-diastolic pressure (mmHg) (*n* = 335)	25 ± 8	25 ± 6	19 ± 7	23 ± 8	18 ± 6	< 0.001
Systolic aortic pressure (mmHg)	146 ± 31	148 ± 28	147 ± 23	150 ± 21	142 ± 23	0.28
Diastolic aortic pressure (mmHg)	67 ± 13	69 ± 12	69 ± 12	69 ± 7	68 ± 11	0.70
Mean aortic pressure (mmHg)	99 ± 16	100 ± 15	99 ± 14	100 ± 12	96 ± 13	0.16
Systemic vascular resistance (Wood units)	22.1 ± 5.4	19.6 ± 5.2	20.9 ± 4.4	18.3 ± 4.2	19.2 ± 4.4	< 0.001
Arterial oxygen saturation (%)	94 (93–96)	95 (93–97)	94 (92–96)	95 (94–96)	96 (95–97)	<0.001
Mixed venous oxygen saturation (%)	63 (58–67)	69 (64–72)	69 (64–71)	71 (67–73)	70 (67–74)	<0.001
Cardiac output (L/min)	4.1 ± 0.8	4.9 ± 1.0	4.6 ± 0.8	5.3 ± 1.0	4.9 ± 0.9	<0.001
Cardiac index (L/min/m^2^)	2.2 ± 0.4	2.5 ± 0.5	2.6 ± 0.5	2.7 ± 0.5	2.7 ± 0.5	<0.001
Stroke volume (mL)	59 ± 18	71 ± 19	68 ± 15	79 ± 16	76 ± 18	<0.001
Stroke volume index (mL/m^2^)	31 ± 9	36 ± 9	37 ± 8	41 ± 8	41 ± 9	<0.001

Data are given as numbers and percentages, mean ± standard deviation, and/or median (interquartile range).

E/e′, ratio of peak early mitral inflow velocity to peak early mitral annular velocity; mPAP, mean pulmonary artery pressure; mPAWP, mean pulmonary artery wedge pressure; sPAP, systolic pulmonary artery pressure; TAPSE, tricuspid annular plane systolic excursion.

### Re-classification

Overall, 156/503 (31%) patients were re-classified by applying the 2022 definition instead of the 2015 definition, i.e. 26 patients were re-classified from no PH to IpcPH, 38 from no PH to pre-capillary PH, 38 from no PH to unclassified PH, 4 from pre-capillary PH to unclassified PH, and 50 patients from IpcPH to CpcPH (*Figure 1*). Patients who were re-classified (*n* = 156) were slightly older and had a slightly lower estimated glomerular filtration rate compared with those who were not (*n* = 347). Key echocardiographic findings and invasive haemodynamics were overall similar in patients who were re-classified and those who were not (data no shown).

### Characteristics of the haemodynamic groups according to the 2022 definition

As expected, the mPAP and the PVR were lower in the CpcPH group according to the less selective 2022 definition compared with the corresponding group according to the 2015 definition because 50 patients with a PVR in the range of 2–3 were re-classified from IpcPH to CpcPH (see Supplementary material online, *Table S3*). At the same time, this contributed to lower mPAP and PVR in the IpcPH group according to the 2022 definition compared with the 2015 definition. The second factor contributing to this phenomenon was the re-classification of 26 patients with mPAWP > 15 mmHg and mPAP between 21 and 24 mmHg from no PH to IpcPH. The number of patients with pre-capillary PH doubled, which was mainly due to the fact that 38 patients with mPAP between 21 and 24 mmHg, mPAWP in the range of 10–15 mmHg, and PVR slightly higher than 2 WU were re-classified from no PH to pre-capillary PH. In this group, the mean mPAWP was borderline, and the mean LVEDP was 19 mmHg. Finally, 42 patients were re-classified into the new category of ‘unclassified PH’. These patients were characterized by a mPAP just above 20 mmHg, an mPAWP between 13 and 15 mmHg, a high-normal PVR (median value 1.7 WU), and the highest stroke volume index among all PH groups (similar to the no PH group) (see Supplementary material online, *Table S3*).

### Prognostic impact of the 2015 vs. 2022 definition

**Figure 2:**
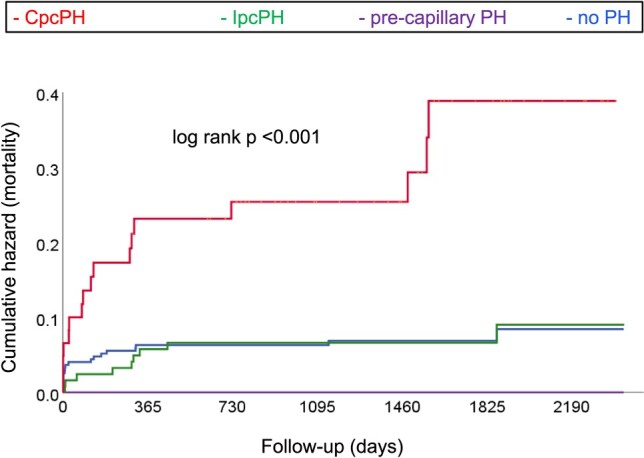
Kaplan–Meier plots showing cumulative events (mortality) for patients with combined pre- and post-capillary pulmonary hypertension (CpcPH), isolated post-capillary PH (IpcPH), pre-capillary PH, and no PH according to the 2015 definition.

**Figure 3:**
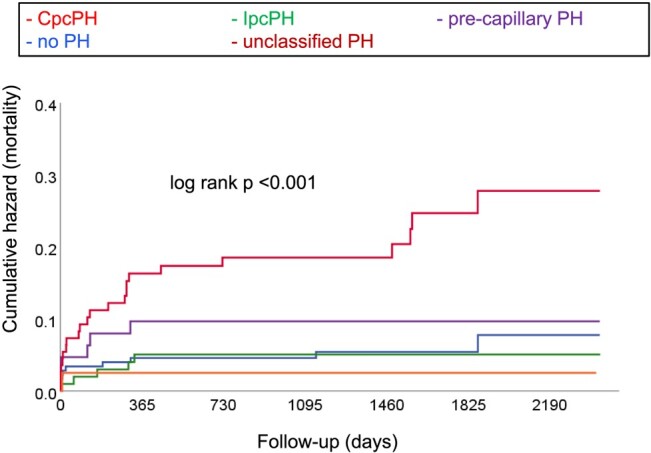
Kaplan–Meier plots showing cumulative events (mortality) for patients with combined pre- and post-capillary pulmonary hypertension (CpcPH), isolated post-capillary PH (IpcPH), pre-capillary PH, unclassified PH, and no PH according to the 2022 definition.

After a median post-AVR follow-up of 1348 (948–1885) days, 45 (9%) deaths had occurred. Causes of death were perioperative/periprocedural within the first 30 days (*n* = 19), cancer (*n* = 4), infection (*n* = 1), cardiovascular (*n* = 4), and unknown (*n* = 17). When using the 2015 definition (*Figure 2*), CpcPH patients experienced a four-fold higher mortality compared with those without PH {hazard ratio [HR] 4.27 [95% confidence interval (95% CI) 2.22–8.22]; *P* < 0.001}. Mortality did not significantly differ among patients with IpcPH, pre-capillary PH, and no PH. Similarly, when applying the 2022 definition (*Figure 3*), CpcPH patients displayed a nearly four-fold higher mortality than to those without PH [HR 3.75 (95% CI 1.79–7.89); *P* < 0.001]. Mortality did not differ between patients with IpcPH [HR 0.87 (95% CI 0.30–2.54); *P* = 0.80], pre-capillary PH [HR 1.67 (95% CI 0.61–4.61); *P* = 0.32], and unclassified PH [HR 0.45 (95% CI 0.06–3.52); *P* = 0.45] compared with the group without PH. Overall, re-classified patients had a similar mortality when compared with those who were not re-classified [HR 1.12 (95% CI 0.60–2.08); *P* = 0.72]. When analysing patients undergoing SAVR (*n* = 361) or TAVR (*n* = 142) separately, mortality in SAVR patients with CpcPH was higher compared with those without PH according to both the 2015 [HR 5.80 (95% CI 2.44–13.78); *P* < 0.001] and 2022 [HR 3.11 (95% CI 1.29–7.52): *P* = 0.01] definitions, whereas there was no difference between the other groups. In patients undergoing TAVR, CpcPH patients had the numerically highest mortality according to both the 2015 [HR 1.66 (95% CI 0.60–4.62); *P* = 0.33] and 2022 [HR 4.92 (95% CI 0.64–37.88); *P* = 0.13] definitions, but due to the limited number of patients in this subgroup, this failed to reach statistical significance (see Supplementary material online, *Figures S1* and *S2*).

### Prognostic impact of key single haemodynamic parameters

**Figure 4:**
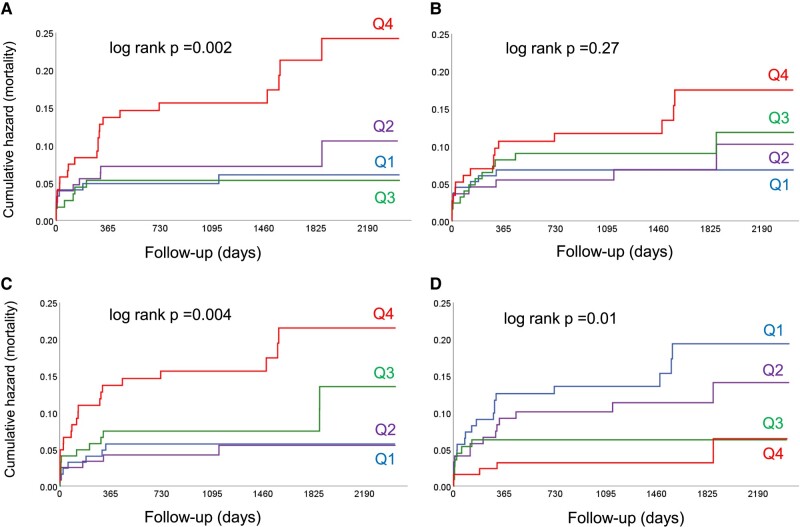
Kaplan–Meier plots showing cumulative events (mortality) according to single haemodynamic parameters. The four categories represent quartiles (Q) for mean pulmonary artery pressure (*A*), mean pulmonary artery wedge pressure (*B*), pulmonary vascular resistance (*C*), and pulmonary artery compliance (*D*).

The prognostic impact of the key single haemodynamic parameters contributing to the PH definitions is illustrated in *Figure 4*. Patients in the highest mPAP quartile had the highest mortality [HR 3.27 (95% CI 1.38–7.58); *P* = 0.007 compared with those in the first quartile (referent)], but mortality did not differ between patients in quartiles 1 to 3, and there was no signal of a dose–effect relationship across these three quartiles (*Figure 4A*). There was no significant mortality difference across mPAWP quartiles, although mortality was numerically highest in patients in the fourth quartile (*Figure 4B*). In contrast, patients in the highest PVR quartile had the highest mortality [HR 3.12 (95% CI 1.33–7.35); *P* = 0.009 compared with those in the first quartile (referent)], and those in the third quartile had intermediate mortality (*Figure 4C*). As shown in *Figure 4D*, patients in the first [HR 3.98 (95% CI 1.49–10.66); *P* = 0.006] and second [HR 2.89 (95% CI 1.04–8.01); *P* = 0.04] PAC quartiles had a significantly higher mortality than those in the fourth quartile (referent), and there was evidence of a dose–effect (PAC–mortality) relationship.

## Discussion

In this first study assessing the impact of the complete 2022 PH definition^1^ in a large cohort of patients with left heart disease due to AS, the following key findings were obtained: (1) approximately one-third of patients were differently classified according to the 2022 vs. 2015 definitions; (2) the proportion of patients with any PH rose from 44% to 64% of the entire population; (3) the re-classification resulted in a substantial increase in the proportion of patients with CpcPH and pre-capillary PH, while the proportion of patients with IpcPH was reduced; (4) 13% of all patients with PH fulfilled the criteria for the new haemodynamic class of ‘unclassified PH’ according to the 2022 definition; (5) patients with CpcPH according to both the 2015 and 2022 definitions had a substantially higher mortality than patients in other PH groups and those without PH; and (6) PVR was the most important PH-defining haemodynamic parameter in terms of prognosis.

Although the 2022 PH definition^15^ has been widely adopted, little data have been published to illustrate the new PH definition’s potential implications.^6–9^ Importantly, the 2022 definition^1^ critically differs from the 2018 World Symposium proposal,^4^ which had been evaluated in some studies, including our previous analysis in the same cohort.^3,16–18^ We had reported that application of the 2018 World Symposium proposal resulted in an 11% re-classification rate in patients with AS when compared with the 2015 definition.^3^ In the present analysis, the increase in the number of patients with any PH and the re-classification rate (2015 → 2022 definition) were much higher, highlighting this difference very clearly. A recent study in a predominantly pre-capillary PH cohort from the UK showed that patients with mildly elevated mPAP (21–24 mmHg) or PVR (>2–≤ 3 WU) according to the 2022 definition had worse survival than those with normal mPAP (<21 mmHg) or PVR (≤2 WU).^5^ The present study focused on post-capillary PH, which represents the vast majority of PH cases in clinical practice. The 2022 definition is less restrictive with regard to the selection of patients with CpcPH [i.e. patients with PVR 2–3 WU (previously labelled IpcPH) are now also included]. There were concerns that this would attenuate the prognostic power of a CpcPH categorization because this group now also includes less advanced cases.^6,10^ In a study including patients with heart failure with preserved left ventricular ejection fraction, a CpcPH categorization according to the 2015 but not according to the 2022 definition was a predictor of increased mortality.^6^ In accordance with two smaller studies among patients with left heart disease,^7,9^ we found a significant shift of patients from the IpcPH to the CpcPH category when applying the 2022 instead of the 2015 definition. In a cohort of patients with a variety of left heart diseases undergoing right heart catheterization for various reasons (*n* = 242), the number of patients with any PH rose from 72 to 76, but the number of patients with CpcPH increased from 4 to 41.^7^ We were able to show that CpcPH patients, according to the 2022 definition, still had a substantially higher mortality than patients without PH or IpcPH, although the hazard ratio was numerically somewhat lower than for CpcPH according to the 2015 definition. Patients with CpcPH had the most severe AS (smallest indexed aortic valve area), which supports the theory that more severe AS with pronounced and sustained elevation of LVEDP and mPAWP leads to pulmonary vascular remodelling and thereby a transition from IpcPH to CpcPH. These patients also had the worst right ventricular function expressed as the lowest tricuspid annular plane systolic excursion, which most likely represents a consequence of high afterload. This represents a constellation of poor right ventricular to pulmonary artery coupling and poor prognosis. We showed that among the PH-defining parameters, PVR was most strongly associated with mortality. This is also in agreement with the recent study by Cardaioli *et al.*,^8^ who found that only patients with increased PVR (i.e. CpcPH and pre-capillary PH according to the 2022 definition) but not IpcPH had worse outcomes than those without PH in patients with severe AS undergoing TAVR. In our study, however, the number of patients with pre-capillary PH nearly doubled with the 2022 definition, but mortality in these patients was not significantly increased, although by definition PVR was higher than 2 WU. Although these patients formally fulfilled the criteria for pre-capillary PH, the mPAWP was borderline (mean 12 mmHg), and the mPAP (mean 25 mmHg) and PVR (median 2.7 WU) were only mildly elevated—by far lower than typically observed in patients with idiopathic pulmonary arterial hypertension. All these patients had severe AS and thereby a strong predisposition for post-capillary PH. We assume that the majority of these ‘pre-capillary PH’ patients had ‘occult post-capillary PH’ after overnight fasting and that only a minority really had two diseases, i.e. severe AS and an unrelated mild pre-capillary PH, e.g. in the context of chronic lung disease. This assumption is supported by the fact that the mean LVEDP in this group was 19 mmHg, as well as by the data of our previous study showing that a volume challenge by means of the contrast applied for coronary angiography leads to an average increase in mPAP and mPAWP by 5 and 4 mmHg, respectively.^19^

We showed that the haemodynamic classification of PH is critically important in terms of prognosis after AVR. Thus, the study indirectly supports the revival of right heart catheterization in patients with valve disease because an exact haemodynamic categorization by echocardiography is not possible. Echocardiography can provide an estimate of the probability of relevant PH and only hints regarding a pre-capillary vs. post-capillary mechanism.^1^ However, the assessment of PVR remains inaccurate. We also showed that the PVR as a continuous variable is by far more relevant in terms of prognosis than the mPAWP and also the mPAP. Therefore, apart from the classification into the CpcPH category, the extent of PVR elevation is critical, particularly after it has now become ‘easier’ to receive a diagnosis of CpcPH with the 2022 PH definition. It has to be taken into account that there is a hyperbolic relationship between PVR and PAC, and a relatively small increase in PVR already represents an important reduction in PAC.^20^ We demonstrated that there is a relatively strong dose–effect relationship for PAC and mortality; therefore, the assessment of PAC apart from PVR in patients with AS undergoing AVR seems worthwhile.

As far as we are aware, this is one of the first two studies also specifically looking at the new haemodynamic category of ‘unclassified PH’, which was introduced because pre-capillary PH is now not only defined by an mPAP > 20 mmHg and an mPAWP ≤ 15 mmHg but also by a PVR > 2 WU and because there are also patients with mPAP > 20 mmHg, mPAWP ≤ 15 mmHg, and PVR ≤ 2 WU.^1^ The World Symposium 2018 proposal has not explicitly addressed this patient group,^4^ but this has now been revised with the 2022 definition.^1^ In the present cohort, this category represented nearly 10% of all patients. As expected from the diagnostic criteria, these patients were characterized by an mPAP just above 20 mmHg, an mPAWP around 15 mmHg, and a PVR close to but below 2 WU. Although these patients formally fulfil the mPAP criterion for PH, filling pressures are still in the normal range, and there is no clear evidence of pulmonary vascular disease based on mPAWP and PVR. Thus, the elevated mPAP is mediated by a relatively high TPG and thus cardiac output. Indeed, this group had a similarly high stroke volume index and a similarly good prognosis as patients without PH. The latter is in line with a recent study among patients undergoing percutaneous edge-to-edge mitral valve repair.^9^ Accordingly, the identification of this PH group with a benign constellation is obviously relevant in different settings to avoid unnecessary concerns from physicians and patients. This observation also supports the liberal use of right heart catheterization in the pre-interventional setting, in particular if echocardiographic findings are ambiguous (e.g. intermediate probability of PH).

The 2022 definition of PH was justified because mPAP > 20 mmHg and PVR > 2 WU are not in the physiological range anymore.^1^ We showed that also in patients with left heart disease and high prevalence of post-capillary PH, the 2022 PH definition represents a prognostically relevant categorization. We also demonstrated that, even in a population with 100% prevalence of significant left heart disease, there is a substantial number of patients fulfilling the haemodynamic criteria for pre-capillary PH or unclassified PH and that it is very important to carefully look at the haemodynamics in detail in these patients to understand and interpret these numbers. This type of analysis is new because most previous studies on the left heart disease setting have not fully applied the 2022 definition but have concentrated on certain PH groups.^7,8^ For example, Güder *et al.*^7^ concentrated on patients with post-capillary PH only, and Cardaioli *et al.*^8^ lumped together pre-capillary PH und CpcPH patients to one single group and have compared them with IpcPH patients and patients without PH. In both studies, there is no information on patients with unclassified PH.

### Limitations

First, the number of patients and the number of events were relatively small, and therefore, power was limited. This is particularly true for the analysis of the subgroups SAVR vs. TAVR. Still, to the best of our knowledge, it is one of the largest cohorts of AS patients undergoing systematic right heart catheterization prior to AVR and one of the first larger-scale studies evaluating the prognostic impact of the 2022 PH definition in a left heart disease population. Second, our data are in part confirmatory of the results recently reported by Cardaioli *et al.*^8^ in a pure TAVR population. However, the advantage of the present analysis is the separation of CpcPH and pre-capillary PH patients and the inclusion of the definition of the unclassified PH group. Third, to assess cardiac output, we have employed the indirect Fick method, which may be subject to error, as oxygen consumption is often inaccurately estimated.^21^ This likely affects all cardiac output-based measurements, including PVR. It must, however, be noted that this technique is routinely used in clinical practice.

## Conclusions

In severe AS, the application of the current PH definition results in a substantially higher number of patients with any PH and CpcPH and leads to a re-classification of one-third of patients. With either definition, CpcPH is associated with a significantly increased post-AVR mortality.

## Lead author biography


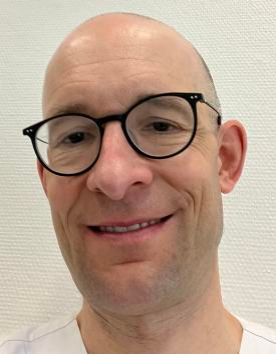


Micha T. Maeder, MD, PhD, is a consultant cardiologist at the Kantonsspital St. Gallen, St. Gallen, Switzerland. Apart from clinical training in Switzerland (Zürich, Basel, Bern, St. Gallen), he had undertaken a research fellowship at the Baker Heart and Diabetes Institute in Melbourne, Australia, and he obtained a PhD degree at the University of Maastricht, Netherlands. His research interests include the pathophysiology of valve disease, heart failure with preserved ejection, and pulmonary hypertension. In clinical practice, he is involved in the non-invasive and invasive management of patients with coronary artery disease, valve disease, pulmonary hypertension, and heart failure.

## Supplementary Material

oeae037_Supplementary_Data

## Data Availability

The analysed data set underlying this manuscript will be shared on reasonable request to the corresponding author.

## References

[1] Humbert M, Kovacs G, Hoeper MM, Badagliacca R, Berger RMF, Brida M, Carlsen J, Coats AJS, Escribano-Subias P, Ferrari P, Ferreira DS, Ghofrani HA, Giannakoulas G, Kiely DG, Mayer E, Meszaros G, Nagavci B, Olsson KM, Pepke-Zaba J, Quint JK, Rådegran G, Simonneau G, Sitbon O, Tonia T, Toshner M, Vachiery JL, Vonk Noordegraaf A, Delcroix M, Rosenkranz S; ESC/ERS Scientific Document Group. 2022 ESC/ERS guidelines for the diagnosis and treatment of pulmonary hypertension. Eur Heart J 2022;43:3618–3731. 36017548 10.1093/eurheartj/ehac237

[2] Galie N, Humbert M, Vachiery JL, Gibbs S, Lang I, Torbicki A, Simonneau G, Peacock A, Vonk Noordegraaf A, Beghetti M, Ghofrani A, Gomez Sanchez MA, Hansmann G, Klepetko W, Lancellotti P, Matucci M, McDonagh T, Pierard LA, Trindade PT, Zompatori M, Hoeper M; ESC Scientific Document Group. 2015 ESC/ERS guidelines for the diagnosis and treatment of pulmonary hypertension: the joint task force for the diagnosis and treatment of pulmonary hypertension of the European Society of Cardiology (ESC) and the European Respiratory Society (ERS): endorsed by: Association for European Paediatric and Congenital Cardiology (AEPC), International Society for Heart and Lung Transplantation (ISHLT). Eur Heart J 2016;37:67–119. 26320113 10.1093/eurheartj/ehv317

[3] Maeder MT, Weber L, Weilenmann D, Chronis J, Joerg L, Pohle S, Haager PK, Brutsche M, Neumann T, Schoch OD, Rickli H. Impact of the new pulmonary hypertension definition on long-term mortality in patients with severe aortic stenosis undergoing valve replacement. Clin Cardiol 2021;44:1276–1285. 34219245 10.1002/clc.23685 PMC8428068

[4] Simonneau G, Montani D, Celermajer DS, Denton CP, Gatzoulis MA, Krowka M, Williams PG, Souza R. Haemodynamic definitions and updated clinical classification of pulmonary hypertension. Eur Respir J 2019;53:1801913. 30545968 10.1183/13993003.01913-2018 PMC6351336

[5] Karia N, Howard L, Johnson M, Kiely DG, Lordan J, McCabe C, Pepke-Zaba J, Ong R, Preiss M, Knight D, Muthurangu V, Coghlan JG. Predictors of outcomes in mild pulmonary hypertension according to 2022 ESC/ERS guidelines: the EVIDENCE-PAH UK study. Eur Heart J 2023;44:4678–4691. 37619574 10.1093/eurheartj/ehad532 PMC10659956

[6] Sera F, Ohtani T, Tamaki S, Yano M, Hayashi T, Nakagawa A, Nakagawa Y, Nakatani D, Yamada T, Yasumura Y, Hikoso S, Yamauchi-Takihara K, Sakata Y; Osaka Cardiovascular Conference (OCVC)—Heart Failure Investigators. Pulmonary hypertension with a precapillary component in heart failure with preserved ejection fraction. Heart 2023;109:626–633. 36543519 10.1136/heartjnl-2022-321565

[7] Guder G, Reiter T, Fette G, Hundertmark M, Frantz S, Morbach C, Störk S, Held M. Diagnosing post-capillary hypertension in patients with left heart disease: impact of new guidelines. Clin Res Cardiol 2023; doi:10.1007/s00392-023-02290-5. PMC12894184 37668664

[8] Cardaioli F, Nai Fovino L, Fabris T, Masiero G, Arturi F, Trevisanello A, Zuccarelli V, Napodano M, Fraccaro C, Continisio S, Tarantini G. Updated definition of pulmonary hypertension and outcome after transcatheter aortic valve implantation. Heart 2023;110:27–34. 37414524 10.1136/heartjnl-2023-322881

[9] Ubben T, Frerker C, Fujita B, Rosenkranz S, Pfister R, Baldus S, Alessandrini H, Kuck K-H, Willems S, Eitel I, Schmidt T. Association of pulmonary hypertension with the outcome in patients undergoing edge-to-edge mitral valve repair. Heart 2024:heartjnl-2023-323473. 10.1136/heartjnl-2023-323473 38388469

[10] Maeder MT . Diagnosing heart failure with preserved ejection fraction with pulmonary vascular disease. Heart 2023;109:578–580. 36543518 10.1136/heartjnl-2022-321984 PMC10086495

[11] Weber L, Rickli H, Haager PK, Joerg L, Weilenmann D, Brenner R, Taramasso M, Baier P, Maisano F, Maeder MT. Haemodynamic mechanisms and long-term prognostic impact of pulmonary hypertension in patients with severe aortic stenosis undergoing valve replacement. Eur J Heart Fail 2019;21:172–181. 30328215 10.1002/ejhf.1322

[12] Reddy YNV, El-Sabbagh A, Nishimura RA. Comparing pulmonary arterial wedge pressure and left ventricular end diastolic pressure for assessment of left-sided filling pressures. JAMA Cardiol 2018;3:453–454. 29590308 10.1001/jamacardio.2018.0318

[13] Gerges M, Gerges C, Lang IM. How to define pulmonary hypertension due to left heart disease. Eur Respir J 2016;48:553–555. 27174881 10.1183/13993003.00432-2016

[14] Vachiery JL, Tedford RJ, Rosenkranz S, Palazzini M, Lang I, Guazzi M, Coghlan G, Chazova I, De Marco T. Pulmonary hypertension due to left heart disease. Eur Respir J 2019;53:1801897. 30545974 10.1183/13993003.01897-2018 PMC6351334

[15] Maron BA, Brittain EL, Hess E, Waldo SW, Baron AE, Huang S, Goldstein RH, Assad T, Wertheim BM, Alba GA, Leopold JA, Olschewski H, Galiè N, Simonneau G, Kovacs G, Tedford RJ, Humbert M, Choudhary G. Pulmonary vascular resistance and clinical outcomes in patients with pulmonary hypertension: a retrospective cohort study. Lancet Respir Med 2020;8:873–884. 32730752 10.1016/S2213-2600(20)30317-9 PMC8219057

[16] Jaafar S, Visovatti S, Young A, Huang S, Cronin P, Vummidi D, McLaughlin V, Khanna D. Impact of the revised haemodynamic definition on the diagnosis of pulmonary hypertension in patients with systemic sclerosis. Eur Respir J 2019;54:1900586. 31196948 10.1183/13993003.00586-2019 PMC7336462

[17] Kovacs G, Zeder K, Rosenstock P, Avian A, Bachmaier G, Douschan P, Foris V, Sassmann T, Olschewsk H. Clinical impact of the new definition of precapillary pulmonary hypertension. Chest 2021;159:1995–1997. 33417899 10.1016/j.chest.2020.11.070

[18] Pfeuffer-Jovic E, Weiner S, Wilkens H, Schmitt D, Frantz S, Held M. Impact of the new definition of pulmonary hypertension according to World Symposium of Pulmonary Hypertension 2018 on diagnosis of post-capillary pulmonary hypertension. Int J Cardiol 2021;335:105–110. 33823213 10.1016/j.ijcard.2021.04.006

[19] Maeder MT, Weber L, Weilenmann D, Haager PK, Joerg L, Rohner F, Ammann P, Chronis J, Rigger J, Rickli H. Impact of a volume challenge on haemodynamics and prognosis in patients with severe aortic stenosis. ESC Heart Fail 2021;8:508–517. 33179419 10.1002/ehf2.13108 PMC7835590

[20] Tedford RJ, Hassoun PM, Mathai SC, Girgis RE, Russell SD, Thiemann DR, Cingolani OH, Mudd JO, Borlaug BA, Redfield MM, Lederer DJ, Kass DA. Pulmonary capillary wedge pressure augments right ventricular pulsatile loading. Circulation 2012;125:289–297. 22131357 10.1161/CIRCULATIONAHA.111.051540 PMC3264431

[21] Gertz ZM, McCauley BD, Raina A, O'Donnell W, Shellenberger C, Willhide J, Forfia PR, Herrmann HC. Estimation of oxygen consumption in elderly patients with aortic stenosis. Catheter Cardiovasc Interv 2014;83:E128–E133. 23704061 10.1002/ccd.25018

