# Supplementary material for: Impact of the 2022 pulmonary hypertension definition on haemodynamic classification and mortality in patients with aortic stenosis undergoing valve replacement
Source: Eur Heart J Open. 2024 May 29;4(3):oeae037. doi: 10.1093/ehjopen/oeae037 (PMC11135639; doi:10.1093/ehjopen/oeae037)
Supplement: oeae037_Supplementary_Data [file oeae037_supplementary_data.docx]

**Supplemental** **Table 1.** Clinical characteristics of the hemodynamic groups according to the 2015 definition

|  | **CpcPH (n=63)** | **IpcPH**  **(n=125)** | **Pre-capillary PH (n=31)** | **No PH**  **(n=284)** | **P value** |
| --- | --- | --- | --- | --- | --- |
| Age (years) | 79±8 | 75±9 | 75±8 | 73±10 | <0.001 |
| Sex (male) | 29 (46%) | 74 (59%) | 18 (58%) | 169 (60%) | 0.26 |
| Body mass index (kg/m^2^) | 26.7±4.5 | 29.4±5.7 | 29.2±5.7 | 27.3±4.7 | <0.001 |
| eGFR (ml/min/1.73m^2^) | 59±21 | 63±20 | 65±19 | 69±17 | <0.001 |
| Hemoglobin (g/l) | 129±17 | 135±19 | 132±26 | 137±16 | 0.007 |
| Albumin (g/l) | 37±3 | 38±4 | 38±4 | 39±5 | <0.001 |
| Sodium (mmol/l) | 137±4 | 138±3 | 138±3 | 138±3 | 0.28 |
| Potassium (mmol/l) | 4.0±0.4 | 4.0±0.5 | 4.0±0.5 | 4.0±0.5 | 0.61 |
| Diabetes | 10 (16%) | 36 (29%) | 8 (26%) | 51 (18%) | 0.05 |
| Insulin-dependent | 3 (5%) | 10 (8%) | 1 (3%) | 8 (3%) | 0.13 |
| Stroke | 4 (6%) | 9 (7%) | 2 (6%) | 15 (5%) | 0.90 |
| Chronic obstructive pulmonary disease | 13 (20%) | 9 (7%) | 8 (26%) | 29 (10%) | 0.003 |
| GOLD stage 1 | 4 | 1 | 0 | 2 |  |
| GOLD stage 2 | 6 | 6 | 5 | 18 |  |
| GOLD stage 3 | 2 | 2 | 3 | 9 |  |
| GOLD stage unknown | 1 | 0 | 0 | 0 |  |
| CPAP therapy | 0 | 1 (1%) | 0 | 1 (<1%) | 0.82 |
| Previous PCI | 5 (8%) | 14 (11%) | 3 (10%) | 24 (8%) | 0.82 |
| Previous CABG | 2 (3%) | 14 (11%) | 4 (13%) | 7 (2%) | 0.001 |
| FEV1 (% predicted) | 77±19 | 83±19 | 77±22 | 91±19 | <0.001 |
| **Heart rhythm*** |  |  |  |  | <0.001 |
| Sinus rhythm | 42 (67%) | 103 (82%) | 29 (94%) | 262 (92%) |  |
| Atrial fibrillation | 17 (27%) | 18 (15%) | 2 (6%) | 13 (5%) |  |
| pacemaker | 4 (6%) | 4 (3%) | 0 | 9 (3%) |  |
| Heart rate (bpm) | 75±15 | 73±14 | 72±16 | 67±11 | <0.001 |
| **Medication** |  |  |  |  |  |
| Oral anticoagulation | 25 (40%) | 30 (24%) | 2 (6%) | 37 (13%) | <0.001 |
| Aspirin | 31 (49%) | 73 (58%) | 23 (74%) | 181 (64%) | 0.07 |
| Loop diuretics | 55 (87%) | 79 (63%) | 15 (48%) | 100 (35%) | <0.001 |
| Betablocker | 30 (48%) | 68 (54%) | 19 (61%) | 121 (43%) | 0.06 |
| ACEI/ARB | 28 (44%) | 76 (61%) | 21 (68%) | 152 (54%) | 0.08 |
| Digoxin | 14 (22%) | 10 (8%) | 2 (6%) | 6 (2%) | <0.001 |
| Spironolactone | 7 (11%) | 8 (6%) | 0 | 10 (4%) | 0.04 |
| B-type natriuretic peptide (ng/l) | 959 (347-1879) | 362 (180-625) | 144 (76-378) | 123 (57-275) | <0.001 |
| **Symptoms** |  |  |  |  |  |
| Dyspnea NYHA class |  |  |  |  | <0.001 |
| I | 5 (8%) | 16 (13%) | 3 (10%) | 77 (27%) |  |
| II | 19 (30%) | 61 (49%) | 14 (45%) | 152 (53%) |  |
| III | 29 (46%) | 44 (35%) | 13 (42%) | 47 (17%) |  |
| IV | 10 (16%) | 4 (3%) | 1 (3%) | 8 (3%) |  |
| STS score | 5.0±3.5 | 3.2±2.2 | 3.0±1.7 | 2.4±1.9 | <0.001 |
| **Mode of AVR** |  |  |  |  | <0.001 |
| Surgical AVR | 32 (51%) | 76 (61%) | 20 (65%) | 233 (82%) |  |
| Transcatheter AVR | 31 (49%) | 49 (39%) | 11 (35%) | 51 (18%) |  |

Data are given as numbers and percentages, mean±standard deviation, or median (interquartile range).

ACEI/ARB = angiotensin converting enzyme inhibitor/angiotensin receptor blocker; AVR = aortic valve replacement; CPAP: continuous positive airway pressure; eGFR = estimated glomerular filtration rate: FEV1 = forced expiratory volume within the first second (percent predicted); GOLD: Global initiative for chronic Obstructive Lung Disease; NYHA = New York Heart Association; STS: Society of Thoracic Surgeons.

*rhythm at the time of cardiac catheterization

**Supplemental** **Table 2.** Data from echocardiography and cardiac catheterization of hemodyamic groups

according to the 2015 classification

|  | **CpcPH (n=63)** | **IpcPH**  **(n=125)** | **Pre-capillary PH (n=31)** | **No PH**  **(n=284)** | **P value** |
| --- | --- | --- | --- | --- | --- |
| **Echocardiography** |  |  |  |  |  |
| Left ventricular end-diastolic diameter (mm) | 48±6 | 47±8 | 46±5 | 45±8 | 0.17 |
| Indexed left ventricular end-diastolic diameter (mm/m^2^) | 27±5 | 24±4 | 25±4 | 25±4 | 0.005 |
| Septal wall thickness (mm) | 14±3 | 13±4 | 12±3 | 12±3 | 0.01 |
| Posterior wall thickness (mm) | 12±3 | 11±3 | 11±3 | 11±2 | 0.11 |
| Left ventricular mass index (g/m^2^) | 134±41 | 112±31 | 108±32 | 105±35 | <0.001 |
| Left ventricular end-diastolic volume index (ml/m^2^) | 49±22 | 46±17 | 42±15 | 43±15 | 0.08 |
| Left ventricular ejection fraction (%) | 51±15 | 53±13 | 58±11 | 61±10 | <0.001 |
| E/e’ | 23±12 | 18±8 | 17±8 | 15.0±7 | <0.001 |
| Left atrial diameter (mm) | 45±7 | 43±7 | 39±6 | 39±7 | <0.001 |
| Indexed left atrial diameter (mm/m^2^) | 25±4 | 22±4 | 21±4 | 21±4 | <0.001 |
| Left atrial area (cm^2^) | 29±9 | 24±6 | 22±4 | 21±6 | <0.001 |
| Indexed left atrial area (cm^2^/m^2^) | 16±4 | 13±3 | 12±3 | 11±3 | <0.001 |
| Left atrial volume index (ml/m^2^) | 59±24 | 45±14 | 39±13 | 38±14 | <0.001 |
| Indexed RV basal diameter (mm/m^2^) | 19±4 | 16±3 | 17±4 | 16±4 | <0.001 |
| Indexed right atrial area (cm^2^/m^2^) | 11±4 | 8±2 | 8±2 | 8±3 | <0.001 |
| Right atrial volume index (ml/m^2^) | 35±17 | 23±10 | 22±9 | 22±11 | <0.001 |
| TAPSE (mm) | 18±5 | 21±5 | 19±4 | 23±5 | <0.001 |
| Estimated sPAP (mmHg) | 51±15 | 41±11 | 47±21 | 34±8 | <0.001 |
| Mean aortic valve gradient (mmHg) | 47±19 | 47±17 | 50±16 | 47±17 | 0.86 |
| Aortic valve area (cm^2^) | 0.70±0.24 | 0.76±0.20 | 0.83±0.22 | 0.82±0.24 | 0.001 |
| Indexed aortic valve area (cm^2^/m^2^) | 0.38±0.13 | 0.39±0.10 | 0.44±0.13 | 0.44±0.10 | 0.001 |
| Mitral regurgitation |  |  |  |  | <0.001 |
| no | 11 (17%) | 38 (30%) | 18 (58%) | 172 (61%) |  |
| mild | 33 (52%) | 74 (59%) | 8 (26%) | 100 (35%) |  |
| moderate | 16 (25%) | 12 (10%) | 4 (13%) | 8 (3%) |  |
| severe | 4 (6%) | 1 (1%) | 1 (3%) | 4 (1%) |  |
| **Coronary artery disease** |  |  |  |  | 0.61 |
| No coronary artery disease | 41 (65%) | 63 (50%) | 13 (42%) | 156 (55%) |  |
| 1-vessel disease | 11 (17%) | 17 (14%) | 6 (19%) | 53 (18%) |  |
| 2-vessel disease | 10 (16%) | 20 (16%) | 4 (13%) | 36 (13%) |  |
| 3-vessel disease | 1 (2%) | 25 (20%) | 8 (26%) | 39 (14%) |  |
| **Invasive hemodynamics** |  |  |  |  |  |
| Mean right atrial pressure (mmHg) | 11±5 | 9±3 | 6±3 | 5±3 | <0.001 |
| Right ventricular end-diastolic pressure (mmHg) | 12±5 | 11±3 | 9±4 | 7±3 | <0.001 |
| sPAP (mmHg) | 65±14 | 47±9 | 45±17 | 30±5 | <0.001 |
| dPAP (mmHg) | 26±7 | 20±5 | 17±6 | 11±4 | <0.001 |
| mPAP (mmHg) | 42±9 | 32±5 | 28±4 | 19±4 | <0.001 |
| mPAWP (mmHg) | 26±7 | 23±5 | 13±2 | 11±4 | <0.001 |
| Transpulmonary gradient (mmHg) | 17±5 | 8±3 | 15±8 | 8±3 | <0.001 |
| Pulmonary vascular resistance (Wood units) | 4.1 (3.3-5.0) | 1.9 (1.3-2.4) | 2.8 (2.3-3.2) | 1.6 (1.2-2.0) | <0.001 |
| Pulmonary artery compliance (ml/mmHg) | 1.5±0.6 | 2.7±1.0 | 3.0±1.2 | 4.1±1.8 | <0.001 |
| Left ventricular end-diastolic pressure (mmHg) (n=335) | 25±7 | 26±8 | 20±7 | 19±7 | <0.001 |
| Systolic aortic pressure (mmHg) | 139±33 | 151±27 | 145±24 | 144±23 | 0.02 |
| Diastolic aortic pressure (mmHg) | 67±13 | 69±12 | 70±11 | 68±11 | 0.45 |
| Mean aortic pressure (mmHg) | 96±16 | 102±15 | 100±13 | 97±13 | 0.02 |
| Systemic vascular resistance (Wood units) | 23.0±5.2 | 20.2±5.2 | 19.7±4.2 | 19.4±4.6 | <0.001 |
| Arterial oxygen saturation (%) | 94 (92-96) | 95 (93-96) | 94 (92-95) | 96 (94-97) | 0.003 |
| Mixed venous oxygen saturation (%) | 62 (56-65) | 67 (63-72) | 68 (60-71) | 70 (67-73) | <0.001 |
| Cardiac output (l/min) | 3.8±0.7 | 4.7±0.9 | 4.9±0.8 | 4.9±1.0 | <0.001 |
| Cardiac index (l/min/m^2^) | 2.1±0.4 | 2.5±0.4 | 2.6±0.5 | 2.6±0.5 | <0.001 |
| Stroke volume (ml) | 53±14 | 68±19 | 71±16 | 75±18 | <0.001 |
| Stroke volume index (ml/m^2^) | 30±8 | 35±9 | 38±8 | 40±9 | <0.001 |

Data are given as numbers and percentages, mean±standard deviation, and/or median (interquartile range).

E/e’ = ratio of peak early mitral inflow velocity to peak early mitral annular velocity, mPAP = mean pulmonary artery pressure; mPAWP = mean pulmonary artery wedge pressure; sPAP = systolic pulmonary artery pressure; TAPSE = tricuspid annular plane systolic excursion.

**Supplemental Table 3.** Comparison of key cardiac catheterization parameters in the different hemodynamic groups according to the 2015 versus the 2022 definition

|  | **CpcPH** | **IpcPH** | **Pre-capillary PH** | **Unclassified PH** | **No PH** |
| --- | --- | --- | --- | --- | --- |
|  | **2015**  **(n=63)** | **2015**  **(n=125)** | **2015**  **(n=31)** | **2015** | **2015**  **(n=284)** |
| mPAP (mmHg) | 42±9 | 32±5 | 28±4 |  | 19±4 |
| mPAWP (mmHg) | 26±7 | 23±5 | 13±2 |  | 11±4 |
| LVEDP (mmHg) | 25±7 | 26±8 | 20±7 |  | 19±7 |
| PVR (WU) | 4.1 (3.3-5.0) | 1.9 (1.3-2.4) | 2.8 (2.3-3.2) |  | 1.6 (1.2-2.0) |
| PAC (ml/mmHg) | 1.5±0.6 | 2.7±1.0 | 3.0±1.2 |  | 4.1±1.8 |
| SVI (ml/m^2^) | 30±8 | 35±9 | 38±8 |  | 40±9 |
|  |  |  |  |  |  |
|  | **2022**  **(n=113)** | **2022**  **(n=101)** | **2022**  **(n=65)** | **2022**  **(n=42)** | **2022**  **(n=182)** |
| mPAP (mmHg) | 39±9 | 28±5 | 25±4 | 22±1 | 16±3 |
| mPAWP (mmHg) | 24±6 | 22±5 | 12±2 | 14±1 | 9±3 |
| LVEDP (mmHg) | 25±8 | 25±6 | 19±7 | 23±8 | 18±6 |
| PVR (WU) | 3.3 (2.5-4.1) | 1.3 (0.9-1.7) | 2.6 (2.2-3.1) | 1.7 (1.6-1.8) | 1.4 (1.0-1.8) |
| PAC (ml/mmHg) | 1.8±0.8 | 3.2±1.3 | 3.1±1.1 | 3.7±1.0 | 4.4±2.1 |
| SVI (ml/m^2^) | 31±9 | 36±9 | 37±8 | 41±8 | 41±9 |

Data are presented as mean±standard deviation or median (interquartile range).

LVEDP = left ventricular end-diastolic pressure, mPAP = mean pulmonary artery pressure, mPAWP = mean pulmonary artery wedge pressure, PAC = pulmonary artery compliance, PVR = pulmonary vascular resistance, SVI = stroke volume index.

**Supplemental Figure 1.** Kaplan Meier plots showing cumulative events (mortality) for patients with combined pre- and post-capillary pulmonary hypertension (CpcPH), isolated post-capillary PH (IpcPH), pre-capillary PH, and no PH according to the 2015 definition in patients undergoing surgical aortic valve replacement (panel A) versus transcatheter aortic valve replacement (panel B).

**Supplemental Figure 2.** Kaplan Meier plots showing cumulative events (mortality) for patients with combined pre- and post-capillary pulmonary hypertension (CpcPH), isolated post-capillary PH (IpcPH), pre-capillary PH, unclassified PH, and no PH according to the 2022 definition in patients undergoing surgical aortic valve replacement (panel A) versus transcatheter aortic valve replacement (panel B).
